# Disruptive Behavior Disorders: Symptoms, Evaluation and Treatment

**DOI:** 10.3390/brainsci12020225

**Published:** 2022-02-07

**Authors:** Annarita Milone, Gianluca Sesso

**Affiliations:** 1 Department of Developmental Neuroscience, IRCCS Stella Maris Foundation, 56128 Pisa, Italy; gsesso@fsm.unipi.it; 2 Department of Clinical and Experimental Medicine, University of Pisa, 56128 Pisa, Italy

## 1. Introduction

Disruptive behavior disorders (DBD) refer to a group of conditions that typically share difficulties in modulating aggressive conducts, self-control, and impulses, with resulting behaviors that constitute a threat to others’ safety and to social norms. Problematic issues with self-control associated with these disorders are commonly first observed in childhood, but may often persist into adolescence and adulthood, or pose a developmental risk for subsequent negative outcomes. The clinical management of DBD in childhood and adolescence has seen great advances in recent years, and research has also focused on identifying early signs, predictors, and risk factors, which may help clinicians to disentangle and subtype the heterogeneous manifestations of BDB. This has allowed significant progress to be made in defining specific developmental trajectories, targeted prevention programs, and timely treatment strategies.

The principal aims of this Special Issue were thus to address three core features of DBD clinical management, namely, its clinical presentations and epidemiologic correlates, including predictors of aggressiveness, gender-specific manifestations, and the role of familiar factors; the multidimensional assessment of callous–unemotional traits, empathic faults, executive dysfunctions, and emotional dysregulation; and the available treatment options, which comprise rehabilitative and pharmacological interventions. In this Special Issue, twelve relevant contributions, including ten original articles, one systematic review, and one study protocol, which provide novel insights for the assessment and treatment of DBD in clinical practice, are reported here (see Table A1).

## 2. Personality Traits and Socio-Environmental Factors as Correlates of Aggressiveness

The study by Robles-Haydar and colleagues [1] primarily aimed to identify the correlates of aggression in non-referred adolescents, hypothesizing a major contribution for moral disengagement in the development of antisocial tendencies. This study examined a set of clinically relevant sociodemographic, personal, and familiar variables, using structural equation models to determine causal relationships. While values of conformity and transcendence seem to inhibit aggressive behaviors, openness, moral disengagement and leadership are among its main predictors, a causal role for gender was also detected. Personality traits and environmental risk factors have been confirmed as well-known essential contributors involved in the development of antisocial conducts in adolescents. These results provide crucial cues, with major relevance in defining primary aims of therapeutic interventions and the prevention of DBD in childhood and adolescence.

Among the predictors of aggressiveness, gender and anxiety were also assessed in a study conducted on 1147 adolescents by Martínez-González and coworkers [2]. In this study, participants watched animated situations of simulated physical aggression against peers and filled out a questionnaire to assess the legitimation of violent behaviors and anxiety levels. While gender was not associated with the behaviors chosen to solve the situation, those adolescents who applied diffusion of responsibility and dehumanization to justify their behavior also showed higher levels of anxiety. Similarly, girls who expected legitimation from their peers presented higher anxiety as well. These results are essential to develop individual and groups of adolescents’ educational programs that improve the awareness of aggressive behaviors’ consequences, modify the way to manage interpersonal conflicts, and change gender-based stereotypes. 

Similar findings were also reported by Catone and collaborators [3] in the Bullying and Youth Mental Health Naples study on cyberbullying behaviors in early adolescence. The aim of the study was to replicate and extend previous findings in a large Italian sample. Conduct problems, callous unemotional traits, traditional bullying and cyberbullying behaviors were assessed by self-rated measures. A significant and specific association between callous unemotional traits and traditional bullying was confirmed, and findings were extended to cyberbullying. This relationship was moderated by conduct problems, while it is independent from gender and age. In particular, males, older adolescents, and those with high scores on conduct problems or CU traits had higher scores on measures of traditional and cyberbullying perpetration. The results of this work can allow one to build psychological and social models of bullying, capable of having a greater impact on prevention and intervention programs.

## 3. Child Temperament, Parenting Style and Family Functioning

Other risk factors for the development of persistent patterns of conduct problems in youths, along with callous unemotional traits, include early parenting style and child temperament. Previous evidence has shown how low parental warmth and fearlessness predict later psychopathic traits in children at increased risk of psychosocial maladjustment. The two-year follow-up study conducted by López-Romero and colleagues [4] on a very large cohort of preschoolers aged 3 to 6 years aimed to disentangle how these factors interact with each other to predict the later occurrence of psychopathic traits. Direct effects from fearlessness to interpersonal and behavioral psychopathic traits were identified, as well as a negative effect of parental warmth, fearless temperament, and their interaction on the development of individual conscience, which in turn mediated the indirect effect of these variables on psychopathic traits. Overall, this study contributed to better understanding the development of child moral adjustment and providing additional insights into effective preventive strategies that may help one to restrain potentially high-risk profiles in early childhood. 

Although parenting style is a key factor in the emergence of conduct problems, research has neglected, so far, the impact of harsh parenting on the siblings of children with DBD. The study performed by Smorti and collaborators [5] indeed aimed to assess parenting styles and siblings’ relationships in sibling dyads of families composed of a DBD child and a non-clinical sibling, and compare them with control families composed of two non-clinical siblings. Sixty-one families were thus recruited and grouped accordingly. Findings indicated differential parenting styles with higher negative parenting toward the DBD child than the sibling, while no difference emerged in sibling relationships within sibling dyads. Externalizing and internalizing problems were also higher in DBD children and their siblings compared to control, confirming psychopathology vulnerability in siblings of children with DBD. Hence, based on these results, the authors of the paper suggested prevention strategies via the inclusion of siblings evaluation in the clinical assessment in DBD children families, and the involvement of parents in multimodal treatments to promote positive parenting, which could have positive effects not only on DBD children, but also for their siblings, in preventing internalizing and externalizing problems.

To further explore the role of family functioning in shaping impulsivity and empathetic abilities in adolescents, the study by Marzilli and coworkers [6] assessed the complex interplay between these variables and the development of antisocial personality traits during emerging adulthood. The study was conducted on a community sample of 350 emerging adults, and found predictive effects of parental control, impulsivity, and empathetic concern in antisocial personality problems. Moreover, motor impulsivity and empathetic concern mediated the relationship between parental control and antisocial personality traits. These results highlight the importance of considering the complex relationship between family functioning, empathy, and impulsivity when evaluating antisocial conducts in youths, and suggest a major protective role, both directly and indirectly, of parental control over behavioral problems. This is crucial for planning more targeted and effective intervention programs that involve parents to ensure the success of prevention goals, but also focus on the promotion of self-control and empathic abilities. 

## 4. Pharmacological Interventions for DBD and Comorbid Conditions

Many DBD patients exhibit an impulsive type of aggressiveness beyond their antisocial behaviors, which is often related to the underlying presence of comorbid ADHD, as one of the most frequently observed in clinical settings. Indeed, in the systematic review conducted by Fantozzi and colleagues [7], the interest was focused on investigating the effects of targeted pharmacological treatments for ADHD on empathic competences, social skills, and aggressiveness in youths. Treatment options included methylphenidate and other stimulant and non-stimulant medications. Thirteen studies were finally included in the review, and data retrieved from individual studies were collected. Ten out of them assessed changes in empathy and the theory of mind, and reported significant improvements in youths treated with either stimulants or nonstimulant drugs. Similarly, seven studies evaluated changes in emotion recognition, though fewer consistent findings were reported. Nonetheless, despite the great heterogeneity in the methodology of the included studies, this systematic review provided evidence for a beneficial effect of medications for ADHD, not only on its core features, but also on cardinal symptoms and drawbacks of DBD. 

Though DBD is among the most common reasons for referral to youth mental health services, the efficacy of therapeutic interventions in clinical practice still remains somewhat unclear. To define more appropriate targets for novel pharmacological treatments for DBD, Balia and collaborators [8], together with the European Commission within the Seventh Framework Program, here proposed the protocol for a multicenter case-control study which will be followed by a single-blind, placebo-controlled, cross-over, randomized acute single-dose medication challenge. This study is part of a larger program aimed to identify the neural, genetic, and molecular underpinnings of aggression and antisocial behaviors in preclinical models and clinical samples, known as the Multidisciplinary Approaches to Translational Research in Conduct Syndromes (MATRICS) project. Aggressive children and adolescents with DBD are here compared to age-matched typically developing controls on a neuropsychological battery, while selected autonomic measures are simultaneously recorded. The acute response to methylphenidate/atomoxetine, risperidone/aripiprazole, or placebo is also examined. 

## 5. Evidence-Based Psychotherapy Approach: The Coping Power Program

Along with pharmacological treatments, evidence-based psychotherapy-oriented interventions are among the cornerstones of the preventive approach for youths with DBD. Among these latter, Coping Power programs have previously shown evidence of clinical effectiveness on children with DBD in reducing the risk of subsequent substance use. In the six-year follow-up study performed by Prof John E. Lochman [9], founder of the Coping Power program and one of the major leading experts in the field of psychoeducational and psychotherapeutic interventions for DBD, a sample of 360 children were randomly assigned to receive either the group or individual program of the Coping Power intervention. The authors observed lower increases in substance use risk for children with low inhibitory control, receiving individual intervention, and for children with higher inhibitory control receiving group intervention. In other words, the level of inhibitory control in aggressive children may help clinicians to tailor specific types of interventions to prevent the risk of later substance use. 

Other formats of the Coping Power intervention program have been conceptualized. The study by Boxmeyer and colleagues [10] was indeed aimed to assess the efficacy of a novel adaptation of the program, known as Mindful Coping Power, on reactive aggression and self-regulation. In a cohort of 102 children, this novel version produced significantly greater improvement in the self-rated measures of emotional, behavioral, and cognitive dysregulation than the classic program; moderate effects were also observed in inhibitory control and breath awareness, as well as in parent-rated measures of attention and social skills, though with milder effects on externalizing problems and reactive aggression. Thus, enhancing effects on more internal embodied experiences are observed with the Mindful Coping Power. 

## 6. Developmental Outcomes and Comorbidities of DBD

Among the severest clinical outcomes of DBD and emotional dysregulation, bipolar disorders with suicidality are often encountered in the developmental trajectory of these adolescents, and often represent a major challenging comorbidity, which is a relevant target for therapeutic purposes. The study by Masi and coworkers [11] aimed to compare suicidality and non-suicidal self-injury (NSSI) in 95 referred bipolar adolescents. The authors found that both were associated with female gender, borderline personality disorder, and symptoms of anxiety and depression; while NSSI was specifically associated with somatic problems, severe suicidal ideation, and attempts were mostly related with adverse life events, bullying, social problems, and feelings of rejection. Thus, both shared and differential features of suicidal ideation and NSSI in adolescence may represent possible targets for early interventions.

Although obsessive compulsive disorder (OCD) and DBD usually exhibit non-overlapping symptoms, they might be theoretically viewed as different developmental outcomes that are derived from a common matrix. On one hand, individuals with OCD show high levels of sense of responsibility and guilt, while, on the other hand, DBD patients lack guilt and are often guided by anti-social purposes. The study by Buonanno and collaborators [12] specifically aimed to investigate the role of forgiveness in responsibility and guilt, and its putative influence by tendencies towards OCD and DBD. Findings with 231 adolescents showed that self-forgiveness predicted high levels of sense of responsibility, while guilt was predicted by both self- and situation-forgiveness. Moreover, the effects of OCD but not DBD symptoms on responsibility and guilt were mediated by self- and situation-forgiveness. 

## 7. Conclusions

Overall, the twelve contributions included in this Special Issue provided novel and fruitful insights from major experts in the field (see Table A1). Indeed, though an increasingly greater amount of studies covering the areas of interest of the present Special Issue has appeared in the literature from all over the world in the past two decades, especially dealing with the heterogeneity of clinical presentations of DBD, their etiopathogenetic factors, and their assessment and treatment options, there are still many questions to be answered. The articles here presented provide novel and helpful elements both for the identification of brand new areas of research, and the development of more effective assessment procedures and treatment strategies in clinical practice. Thus, we would like to express our deepest gratitude to all authors who contributed to this Special Issue “Disruptive Behavior Disorders: Symptoms, Evaluation and Treatment”, and the reviewers, for their dedicated time and help in improving the quality of published manuscripts.

## Appendix A

**Table A1:** Summary of the included studies.

Ref	Authors	Country	Institution	Type
[1]	Robles-Haydar CA, Martínez-González MB, Flórez-Niño YA, Ibáñez-Navarro LM, Amar-Amar JJ.	Colombia	Universidad de la Costa, Universidad del Corte	Article
[2]	Martínez-González MB, Turizo-Palencia Y, Arenas-Rivera C, Acuña-Rodríguez M, Gómez-López Y, Clemente-Suárez VJ.	Colombia, Spain	Universidad de la Costa, Universidad Europea de Madrid	Article
[3]	Catone G, Almerico L, Pezzella A, Riccio MP, Bravaccio C, Bernardo P, Muratori P, Pascotto A, Pisano S, Senese VP.	Italy	Suor Orsola Benincasa University, University of Campania “Luigi Vanvitelli”, Federico II University, Santobono-Pausilipon Children Hospital, IRCCS Stella Maris Foundation	Article
[4]	López-Romero L, Cutrín O, Maneiro L, Domínguez-Álvarez B, Romero E.	Spain, Netherlands	Universidade de Santiago de Compostela, Leiden University	Article
[5]	Smorti M, Inguaggiato E, Vezzosi L, Milone A.	Italy	University of Pisa, IRCCS Stella Maris Foundation	Article
[6]	Marzilli E, Cerniglia L, Cimino S.	Italy	University of Rome “La Sapienza”, International Telematic University Uninettuno	Article
[7]	Fantozzi P, Sesso G, Muratori P, Milone A, Masi G.	Italy	IRCCS Stella Maris Foundation, University of Pisa	Systematic Review
[8]	Balia C, Carucci S, Milone A, Romaniello R, Valente E, Donno F, Montesanto A, Brovedani P, Masi G, Glennon JC, Coghill D, Zuddas A, The Matrics Consortium.	Italy, Ireland, Netherlands, Australia	University of Cagliari, “Cao” Pediatric Hospital, ARNAS “Brotzu” Hospital Trust, IRCCS Stella Maris Foundation, University College Dublin, Radboud University, University of Dundee, Murdoch Children’s Research Institute of Melbourne, University of Melbourne	Study Protocol
[9]	Lochman JE, Boxmeyer CL, Bui C, Hakim E, Jones S, Kassing F, McDonald K, Powell N, Qu L, Dishion T.	USA	University of Alabama, Arizona State University	Article
[10]	Boxmeyer CL, Miller S, Romero DE, Powell NP, Jones S, Qu L, Tueller S, Lochman JE.	USA	University of Alabama, University of Texas at San Antonio	Article
[11]	Masi G, Lupetti I, D’Acunto G, Milone A, Fabiani D, Madonia U, Berloffa S, Lenzi F, Mucci M.	Italy	IRCCS Stella Maris Foundation	Article
[12]	Buonanno C, Iuliano E, Grossi G, Mancini F, Stendardo E, Tudisco F, Pizzini B.	Italy	School of Cognitive Psychotherapy, InMovement Center, University of Rome “Guglielmo Marconi”, University of Campania “Luigi Vanvitelli”	Article
